# Mesothelioma as a rapidly developing Giant Abdominal Cyst

**DOI:** 10.1186/1477-7819-10-277

**Published:** 2012-12-20

**Authors:** Dinesh Vyas, Kerent Pihl, Srinivas Kavuturu, Arpita Vyas

**Affiliations:** 1 Department of Surgery, College of Human Medicine, Institute of International Health, Michigan State University, 1200 East Michigan Avenue, Suite 655, Lansing, MI, 48912, USA; 2 Michigan State University, 1200 East Michigan Avenue, Suite 655, Lansing, MI, USA; 3 Department of Surgery, College of Human Medicine, Michigan State University, 1200 East Michigan Avenue, Suite 655, Lansing, MI, 48912, USA; 4 Department of Pediatrics, College of Human Medicine, Michigan State University, Lansing, MI, 48912, USA

## Abstract

The benign cystic mesothelioma of the peritoneum is a rare lesion and is known for local recurrence. This is first case report of a rapidly developing massive abdominal tumor with histological finding of benign cystic mesothelioma (BCM). We describe a BCM arising in the retroperitoneal tis[sue on the right side, lifting ascending colon and cecum to the left side of abdomen. Patient was an active 58-year-old man who noticed a rapid abdominal swelling within a two month time period with a weight gain of 40 pounds. Patient had no risk factors including occupational (asbestos, cadmium), family history, social (alcohol, smoking) or history of trauma. We will discuss the clinical, radiologic, intra-operative, immunohistochemical, pathologic findings, and imaging six months after surgery. Patient has no recurrence and no weight gain on follow up visits and imaging.

## Case report

**Figure 1:**
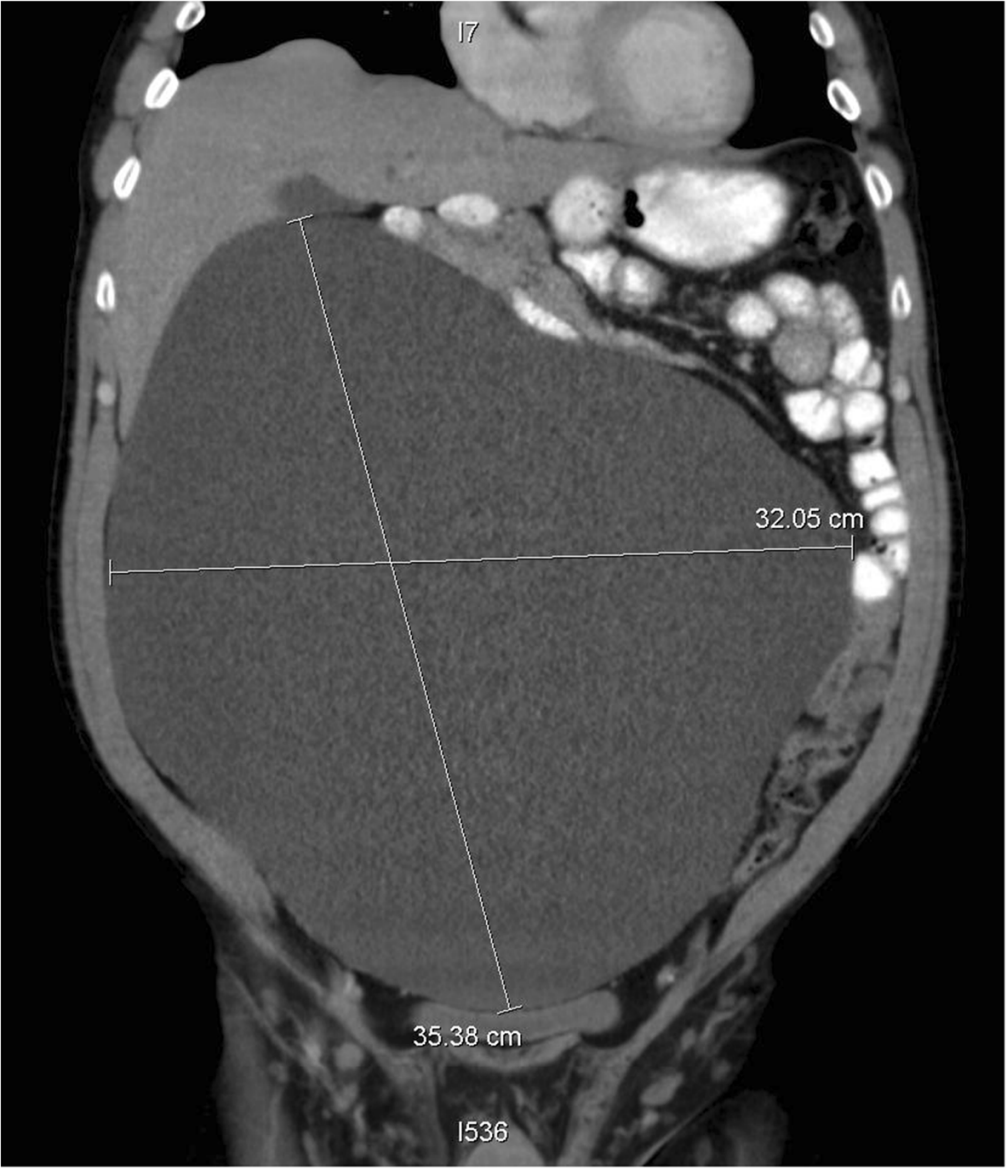
Pre-operative computer tomography (CT) scan images (axial, coronal and sagittal views of the giant cyst).

We describe a healthy and active 58-year-old man, who four months prior to presentation noticed that he was gaining weight, in spite of participating in aerobic exercise daily and having no change in diet. Three months prior to presentation the patient began noticing an increase in abdominal girth, stating that it, ‘felt like a large ball in my belly. The patient began to develop occasional abdominal discomfort, which slowly increased in pain and frequency. The patient also developed symptoms of constipation and severe gastroesophageal reflux, for control of which, he began taking laxatives and a proton pump inhibitor. He eventually saw his primary care physician and had a computer tomography (CT) scan done, showing a large spherical cystic mass in the abdomen measuring 32 cm by 25 cm (Figure 1). The CT scan findings in short were of a massive uniloculated retroperitoneal cyst seen non- communicating to the pleura, or any other abdominal viscera and occupying nearly the entire abdominal space, displacing his entire bowel to the patient’s left, with the cecum and ascending colon located anterior and left of the midline. The mass spanned from the pelvis inferiorly to the liver hilum superiorly. The liver and stomach were compressed. The patient was referred to a surgeon and the recommendation was made to proceed with surgery after complete evaluation. He has no risk factors related to the mesothelioma (exposure to asbestos or any virus) or any malignancy. We checked other risk factors including occupational (exposure to silica or cadmium), family history, social (alcohol intake and smoking) or history of trauma.

**Figure 2:**
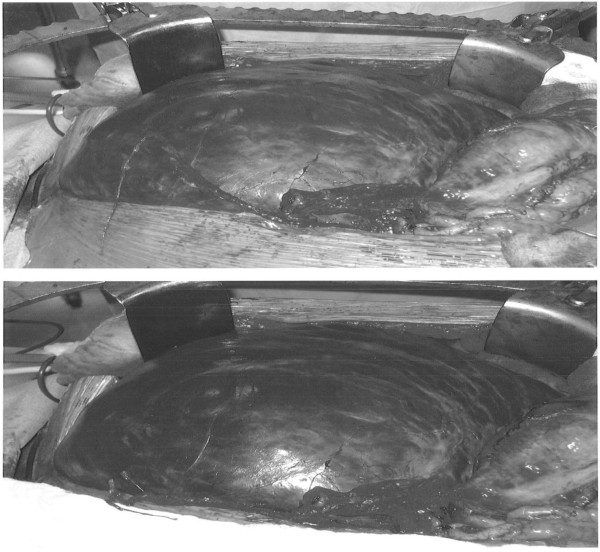
Intra-operative image of the cyst.

**Figure 3:**
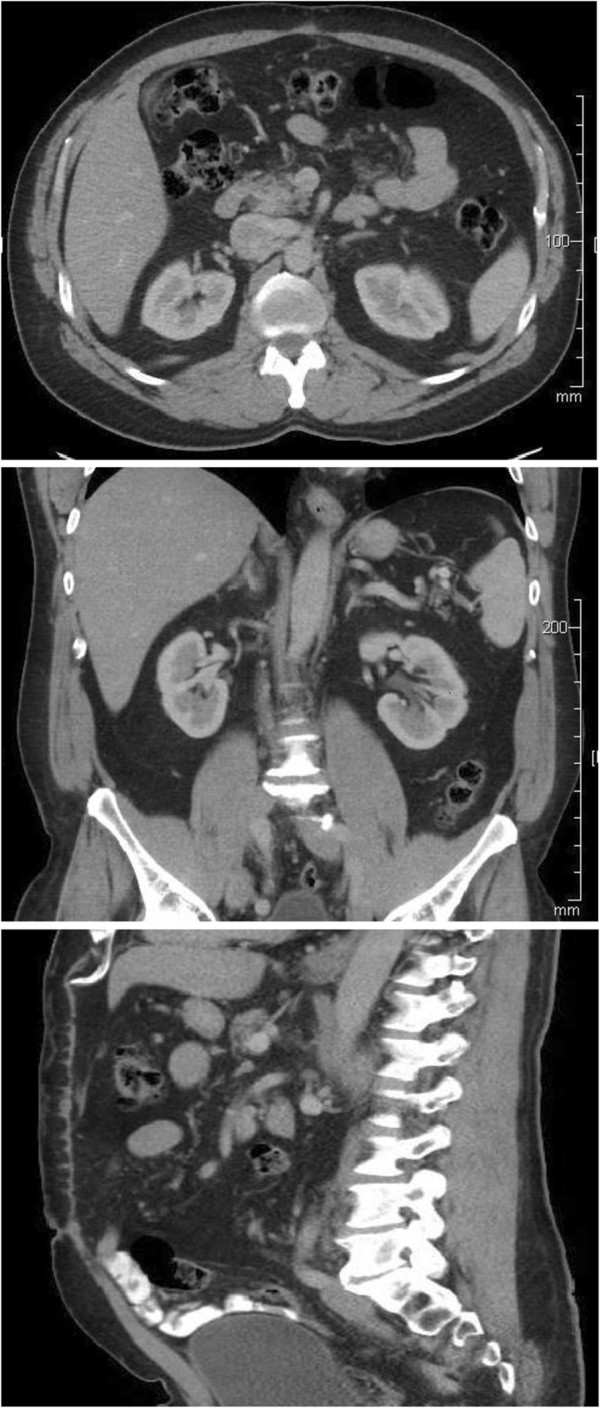
Post-operative computed tomography (CT) (axial, coronal and sagittal images).

In the operating room, after a midline laparotomy, a massive retroperitoneal cyst was discovered to span nearly the entire abdominal width, displacing his entire bowel to the patient’s left, with the cecum and ascending colon located anterior and left of the midline. The mass spanned from the pelvis inferiorly to the liver hilum superiorly. The colon was dissected away from the mass and two layers of pseudomembrane were dissected off the anterior surface of the cyst. The mass was then dissected off the retroperitoneal organs using blunt dissection as there was only loose areolar tissue connecting the structures (Figure 2). An initial attempt was made to remove the entire cystic mass intact, however the thin cyst wall ruptured and 15 liters of serous fluid was aspirated. There was no evidence of a tract or origin to the cystic structure. Grossly the mass appeared uniloculated with a smooth thin wall and straw-colored fluid. Microscopically, the sections showed a benign multicystic lesion lined by cuboidal cells which stained positive for pancytokeratin, calretinin, and D2-40 and negative for CD34. We are following our patient every 3 months and performed CT imaging before every visit for the first two visits, and then before every fourth visit. CT from the second clinical visit shows no recurrence and the patient has returned to his normal active state (Figure 3). There is no recurrence on post-operative visits.

## Discussion

Mesothelioma is a rare form of cancer seen in mesothelium, the cellular lining covering many internal organs. The most common site is the pleura, followed by the peritoneum and the pericardium. Retroperitoneal cystic masses are a rare occurrence with a combined incidence of 1:106,400 to 1:250,000 [1-3] when also including mesenteric cystic masses. The differential diagnosis is vast, including cystic lymphangioma, mucinous cystadenoma, cystic teratoma, cystic mesothelioma, mullerian cyst, epidermoid cyst, tailgut cyst, bronchogenic cyst, cystic changes in a solid neoplasm, pseudomyxoma retroperitonei, perianal mucinous carcinoma, pancreatic pseudocyst, lymphocele, urinoma, hematoma [4], cystadenoma of mesonephric origin, and cavernous hemangioma [5]. Due to the rarity of these conditions, similarity of patient presentation and comparable features on imaging, the correct pre-operative diagnosis may be difficult to attain [4].

Benign cystic mesothelioma is also a rare entity with most information coming from individual case reports. It was first described by Henke in 1889 as a ‘multiple cystic lymphangioma like tumor [6], determined to be of mesothelial origin by Mennemeyer and Smith in 1979 [7], and given the name ‘benign cystic mesothelioma in 1980 by Moore, *et al*. [8]. The etiology remains unclear and there is no association with asbestos exposure, unlike malignant mesothelioma. There have been rare reports of transformation to low-grade malignant mesothelioma, but it is generally classified as a benign process [9]. There is a 40 to 55% recurrence rate in female patients and a 33% recurrence rate in male patients [10]. The clinical, gross, and histologic findings in our case of BCM were similar to those reported in the literature [11-14].

The common presenting features are those of a massive lesion with effects including abdominal pain, fullness, distention, and intestinal obstruction, and if allowed to enlarge significantly, weight gain. Physical examination may show abdominal tenderness, distention, or a palpable mass. Imaging modalities that can be utilized include ultrasonography (US), CT, or magnetic resonance imaging (MRI). CT is often the most useful as it provides sectional images of the abdominal and retroperitoneal compartments, and gives information in relation to nearby organs, which helps to determine feasibility of resection, and clues to cystic contents [15]. Surgery, with complete enucleation of the cyst to prevent recurrence and possible malignant transformation remains the mainstay of treatment [16], which can be performed with either a laparotomy or via laparoscopy [17]. Aspiration of the cyst alone may be beneficial to relieve symptoms, but due to high recurrence rates is not a definitive treatment and is rarely beneficial in the pre-operative diagnosis.

Imaging has limitations in diagnosing BCM and the definitive diagnosis is based on histological studies. Pre-operative diagnosis is often incorrect and post-operative diagnosis is made with a combination of microscopy and immunohistochemistry or electron microscopy. Histopathologically, BCM demonstrates a multicystic lesion lined by a single layer of cuboidal cells of mesothelial origin separated by a delicate fibromuscular stroma [18]. Electron microscopy shows characteristic cuboidal cells with innumerable microvilli on the luminal surfaces as well as intercellular bridges with desmosomes distinguishing it from the endothelial cells of lymphangioma [8]. Immunohistochemistry will be positive for cytokeratin and mesothelial origin markers calretinin and D2-40. It will be negative for the lymphoid marker CD34. The mesothelial epithelium can be confirmed by its immunopositivity for calretinin, pancytokeratin, cytokeratin 5/6, HBME1, and EMA and the negativity for CEA, CA-125, factor VIII-related antigen, and CDX-2 [13].

There is a role for using fine needle aspiration with immunohistochemical stain for definitive diagnosis but there is a concern is some parts of world about hydatid cysts and anaphylaxis from cyst rupture [19,20]. A cystic lymphatic malformation can be confused with BCM because of the mutually similar gross appearance and the prevalence of lymphatic malformations. Other differential diagnosis can be a well-differentiated papillary mesothelioma with a cystic form of malignant mesothelioma [11]. Complete surgical excision, is the treatment of choice, even though some centers advocate an aggressive surgical treatment including cytoreductive surgery with peritonectomy procedures and hyperthermic intraperitoneal chemotherapy. The jury is divided on the choice of treatment for recurrent disease, from selective and limited surgery for the symptoms of intestinal obstruction or other functional abnormalities and severe abdominal pain, to cytoreductive surgery with peritonectomy procedures and hyperthermic intraperitoneal chemotherapy [21].

Due to the rarity of BCM there are no established follow-up or post-operative imaging guidelines. With the relatively high recurrence rates of BCM, follow-up is advised, especially if complete enucleation could not be accomplished. We recommend CT scan follow-up at least yearly for 5 years, and we are performing this every 3 months for first year and then yearly due to intra-operative spillage.

## Authors’ contributions

Study conception and design: VD, KP. Acquisition of data: VD, KP, KS. Analysis and interpretation of data: VD, KP, VA. Drafting of manuscript: VD, KP, VA. All authors read and approved the final manuscript.
